# Natural antiviral compound silvestrol modulates human monocyte‐derived macrophages and dendritic cells

**DOI:** 10.1111/jcmm.15360

**Published:** 2020-05-06

**Authors:** Leonard Blum, Gerd Geisslinger, Michael J. Parnham, Arnold Grünweller, Susanne Schiffmann

**Affiliations:** ^1^ Pharmazentrum Frankfurt/ZAFES Institute of Clinical Pharmacology Goethe‐University Hospital Frankfurt Frankfurt am Main Germany; ^2^ Branch for Translational Medicine and Pharmacology TMP Fraunhofer Institute for Molecular Biology and Applied Ecology IME Frankfurt am Main Germany; ^3^ Institute of Pharmaceutical Chemistry Philipps‐University Marburg Marburg Germany

**Keywords:** antiviral, cytokines, eIF4A, energy metabolism, immune modulation, RNA viruses, rocaglate

## Abstract

Outbreaks of infections with viruses like Sars‐CoV‐2, Ebola virus and Zika virus lead to major global health and economic problems because of limited treatment options. Therefore, new antiviral drug candidates are urgently needed. The promising new antiviral drug candidate silvestrol effectively inhibited replication of Corona‐, Ebola‐, Zika‐, Picorna‐, Hepatis E and Chikungunya viruses. Besides a direct impact on pathogens, modulation of the host immune system provides an additional facet to antiviral drug development because suitable immune modulation can boost innate defence mechanisms against the pathogens. In the present study, silvestrol down‐regulated several pro‐ and anti‐inflammatory cytokines (IL‐6, IL‐8, IL‐10, CCL2, CCL18) and increased TNF‐α during differentiation and activation of M1‐macrophages, suggesting that the effects of silvestrol might cancel each other out. However, silvestrol amplified the anti‐inflammatory potential of M2‐macrophages by increasing expression of anti‐inflammatory surface markers CD206, TREM2 and reducing release of pro‐inflammatory IL‐8 and CCL2. The differentiation of dendritic cells in the presence of silvestrol is characterized by down‐regulation of several surface markers and cytokines indicating that differentiation is impaired by silvestrol. In conclusion, silvestrol influences the inflammatory status of immune cells depending on the cell type and activation status.

## 1 INTRODUCTION

Over the last few years, we have had to face several severe virus‐mediated disease outbreaks like the current worldwide Sars‐CoV‐2 pandemic, the Ebola virus outbreak in West Africa and the Zika virus outbreak in South America. The treatment options for virus‐mediated diseases are limited, so well‐tolerated as well as efficient antiviral therapies are urgently needed.^1^ The use of the natural compound silvestrol is a promising new broad‐spectrum approach for the treatment of viral infections. Silvestrol can be isolated from the plants of the genus *Aglaia* ^2^ and was initially described in the field of cancer research where it showed potent anti‐tumour activity in vivo and in vitro.^3^, ^4^, ^5^ The effects of silvestrol are based on the highly specific inhibition of the ATP‐dependent DEAD‐box RNA helicase eIF4A.^6^, ^7^ Several viruses rely on this host factor for the translation of their mRNAs. The targeting of host factors has advantages, like a decreased risk of escape mutations by the virus,^8^ but also presents difficulties compared to viral targets, such as possible pleiotropic side effects.^9^ However, the inhibition of eIF4A by silvestrol appears to be highly specific which should minimize the risk of side effects. Silvestrol showed, moreover, a broad range of potent antiviral effects on different RNA viruses. For instance, silvestrol inhibits the replication of Coronaviruses,^10^ Ebola virus,^11^ Zika virus^12^ as well as subtypes of Picornaviruses,^10^ Chikungunya virus^13^ and reduces the release of hepatitis E virus infectious particles.^14^

Some intracellular bacterial pathogens have developed sophisticated strategies to prevent M1‐like polarization of macrophages, thereby altering microbicidal mechanisms or driving the polarization towards an M2 phenotype to reduce the defensive host inflammatory response.^15^ In this respect, it is noteworthy that several antibiotics are able to activate the host immune system and thereby increase immune defence mechanisms independently of the direct drug impact on the microorganism.^16^ Such modulation of the immune system can broaden the drug efficacy profile boosting innate host defence mechanisms and thereby increasing pathogen clearance while reducing unwanted tissue damage by extenuated inflammation. Because silvestrol regulates the translation of the mRNA encoding the signal transducer and activator of transcription 1 (STAT1) transcription factor^17^ that promotes innate and adaptive immune responses,^18^ we speculated that silvestrol possibly interacts with the host immune system and thereby bolsters its antipathogenic effect and/or promotes resolution of inflammation and tissue damage.

Most infectious diseases are accompanied by local inflammation and accumulation of various immune cells, such as monocytes, macrophages and dendritic cells, at the site of infection, where they release a broad range of cytokines, chemokines and lipid mediators, which facilitate pathogen clearance. To minimize the tissue damage resulting from exaggerated inflammation, well‐timed resolution is essential. Macrophages play a major role in initiation and resolution of inflammation. They initiate the local inflammation through release of cytokines such as interleukin (IL)‐1β, interferon (IFN)‐γ, IL‐23 and tumour necrosis factor (TNF)‐α and recruit further pro‐inflammatory immune cells by the release of chemokines (eg CC‐chemokine ligand (CCL)2, C‐X‐C motif chemokine (CXCL)10, IL‐8).^19^ Macrophages and dendritic cells recognize microbial carbohydrates and mediate phagocytosis via pattern recognition receptors such as CD206 or CD209.^20^, ^21^ Thereby, macrophages ingest invading pathogens and present pathogenic peptides via HLA‐DR to T cells for the activation of the acquired immune system. M2 macrophages also release cytokines such as IL‐10 to support the process of tissue healing and remodelling^22^ and chemokines such as CCL18 or CCL17 to recruit anti‐inflammatory T_H_2 and T_reg_ cells.^19^, ^23^, ^24^ Dendritic cells are mainly responsible for the presentation of antigens, the control of the antigen‐specific response of T cells and the intensity of the inflammatory process. Activation of dendritic cells induces their expression of co‐stimulation molecules (eg CD80, CD86) and HLA‐DR.

In the present study, we investigated the immunomodulatory effects of the natural compound silvestrol on human monocyte‐derived macrophages (MdMs) and dendritic cells (MdDCs). For this purpose, we isolated CD14^+^ cells from fresh human blood samples and examined the impact of silvestrol on cell viability, cell‐type–specific surface markers, released cytokines and energy metabolism during differentiation and polarization.

## 2 MATERIALS AND METHODS

### 2.1 Cells and reagents

Human monocytes, macrophages and dendritic cells were cultured in RPMI1640‐Glutamax medium supplemented with 1% penicillin/streptomycin 10% FCS at 37°C in 5% CO_2_ atmosphere. Buffy coats from healthy donors were obtained freshly from DRK‐Blutspendedienst. Orangu assay was purchased from Cell guidance systems. Human FcR Blocking Reagent, human CD14 MicroBeads, human granulocyte macrophage colony‐stimulating factor (GM‐CSF), macrophage colony‐stimulating factor (M‐CSF), bovine serum albumin (BSA), IL‐4 and all antibodies for surface staining were purchased from Miltenyi Biotec. Cytometric bead array was purchased from BD Biosciences. ELISA for CCL18 and CCL17 was purchased from BosterBio and BioLegend, respectively. Accutase^®^ solution and Biocoll were purchased from Merck. EDTA was purchased from Sigma‐Aldrich. Silvestrol (purchased from the Sarawak Biodiversity Centre, Kuching, Borneo at a purity of >99%) was dissolved in DMSO and further diluted in medium (c_stock_ = 6 mmol/L, maximal DMSO concentration during experiments 0.000083% v/v).

### 2.2 Isolation of human CD14^+^ cells

Human peripheral blood mononuclear cells (PBMC) were isolated from fresh buffy coats by density gradient. Therefore, 25 mL of blood from healthy donors was mixed 1:1 with Hank's balanced salt solution (HBSS, Thermo Fisher Scientific) and layered over 15 mL of Biocoll (Merck) in Sep‐Mate™‐50 Tubes (Stemcell Technologies). After centrifugation (1200 g, 10 minutes, RT), PBMCs from the interphase were isolated and washed four times with 2 mmol/L EDTA/PBS. Cell count was determined using MACSQuant® Analyzer 10 flow cytometer (Miltenyi Biotec). CD14^+^ cells were isolated with human CD14 MicroBeads from Miltenyi Biotec according to the protocol. Briefly, defined amounts of cells were dissolved in 0.5% BSA/2 mmol/L EDTA/PBS and incubated with 25% (v/v) human CD14 MicroBeads for 15 minutes at 4°C. After incubation, the magnetic labelled cells were separated from unlabelled cells via magnetic cell separation (MACS) with LS columns and cell counts were determined using MACSQuant^®^ Analyzer 10.

### 2.3 Cell viability assay

For determination of the cell viability, Orangu assay was used according to the manufactory guidelines. Briefly, 1 × 10^5^ monocytes were seeded in 96‐well plates and incubated with various concentrations of silvestrol, with vehicle (DMSO) or were left untreated. After 30 minutes incubation (37°C, 5% CO_2_), 10 ng/mL GM‐CSF and 10 ng/mL IL‐4 were added to differentiate MdDCs or 10 ng/mL GM‐CSF to differentiate MdMs. For MdMs, the medium was completely refreshed after 3 days of incubation. After 48 hours (monocytes), 5 days (MdDCs) or 7 days (MdMs) 10 µL of Orangu™ cell counting solution was added to the wells and incubated for 120 minutes (37°C, 5% CO_2_). Absorbance was measured at 450 nm with 650 nm as reference using EnSpire Plate Reader (PerkinElmer). Sample values were corrected with the background wells containing only medium without cells. Absorbance from treated cells was set in correlation to untreated cells.

### 2.4 Differentiation of human macrophages and dendritic cells

For differentiation of MdMs or MdDCs, 0.5 × 10^6^ or 0.9 × 10^6^ CD14^+^ cells/well, respectively, were cultivated in the presence of different concentrations of silvestrol or vehicle (DMSO) in 48‐well plates. After pre‐incubation with silvestrol or DMSO for 30 minutes, stimulants for differentiation were added: 10 ng/mL GM‐CSF for MdMs and 10 ng/mL GM‐CSF and 10 ng/mL IL‐4 for MdDCs. MdDCs were incubated for 5, MdMs for 7 days (37°C, 5% CO_2_). MdM medium with growth factors and silvestrol was completely refreshed after 3 days. After differentiation, cells were centrifuged (300 g, 5 minutes, RT) and supernatant was stored for cytokine and chemokine detection at −80°C. Cells were washed with PBS, harvested with Accutase^®^ solution (15 minutes, 37°C, 5% CO_2_) and cell count was determined using MACSQuant^®^ Analyzer 10.

### 2.5 Polarization of human macrophages

For polarization of MdMs, 7.5 × 10^6^ CD14^+^ cells were seeded in T‐75 flasks (Thermo Fisher Scientific) and stimulated with 10 ng/mL human GM‐CSF (M1‐polarization) or 50 ng/mL human M‐CSF (M2‐polarization). After 7 days of incubation (37°C, 5% CO_2_), cells were washed with PBS and incubated with 5 mL Accutase® solution (15 minutes, 37°C, 5% CO_2_). Afterwards, 15 mL medium was added, cells were scraped off and cell count was determined using MACSQuant^®^ Analyzer 10. In 48‐well plates, 5 × 10^5^ cells/well were seeded and after 30 minutes of pre‐incubation (37°C, 5% CO_2_) with silvestrol or vehicle (DMSO), 20 ng/mL human IFN‐γ (M1‐polarization) or 10 ng/mL human IL‐4 (M2‐polarization) was added. After 24 hours (M2‐polarization) or 48 hours (M1‐polarization), cells were harvested with Accutase^®^ solution (15 minutes, 37°C, 5% CO_2_) and cell count was determined using MACSQuant^®^ Analyzer 10.

### 2.6 Activation of human dendritic cells

For differentiating monocytes to dendritic cells, 1.5 × 10^7^ isolated CD14^+^ cells were seeded in T‐75 flasks with 50 ng/mL of human GM‐CSF and 50 ng/mL of human IL‐4. After 5 days of differentiation without silvestrol, cells were harvested and seeded in triplicate, with 0.9 × 10^6^ cells/well in 48‐well plates. Silvestrol (0.5‐5 nmol/L) or vehicle (DMSO) was added, and after 30 minutes of pre‐incubation (37°C, 5% CO_2_), 5 ng/mL human TNF‐α, 5 ng/mL human IL‐6, 5 ng/mL human IL‐1β, and 500 ng/mL PGE_2_ were added. Cells were incubated for 24 hours (37°C, 5% CO_2_) and harvested for analysis via flow cytometry. Supernatants were stored at −80°C for cytokine analysis.

### 2.7 Flow cytometry

For analysis of the surface markers, 1.5‐2 × 10^5^ cells of each sample were blocked with human FcR Blocking Reagent (15 minutes, 4°C). To discriminate living and dead cells, Zombie Aqua™ Fixable Viability Kit (1:500 dilution, BioLegend) was used according to the manufactory protocol. Afterwards, samples were stained with up to 7 µL of a mix of different surface marker antibodies (15 minutes, 4°C) and 250 µL of 10% FCS/PBS was added. After centrifugation (300 g, 5 minutes, 4°C), samples were resuspended in 100 µL PBS and measured with MACSQuant^®^ Analyzer 10 flow cytometer. Geometric mean of the fluorescent intensity was calculated using FlowJo software v10 (Treestar). Fold induction of surface marker expression was calculated using the DMSO‐treated cells as control.

### 2.8 Cytometric bead array/ELISA

For determination of cytokine/chemokine concentrations in the supernatant of differentiated/polarized humane immune cells, cytometric bead array (BD Biosciences) for IL‐10, IL‐8, IL‐6, IL‐1β, TNF‐α and CCL2 or ELISA for CCL18, CCL17 and IL‐23 was performed. The cytometric bead array and the ELISA were performed according to the manufactory protocol.

### 2.9 Determination of energy metabolism

For the analysis of the extracellular acidification rate (ECAR) and the oxygen consumption rate (OCR) for human monocytes, macrophages and dendritic cells, the Seahorse XFe96 FluxPak (Agilent) was used as recommended by the manufacturer. CD14^+^ cells were isolated and human monocytes were cultivated for 48 hours without further differentiation factors while macrophages and dendritic cells were differentiated as described before. All cells were cultivated in the presence of 5 nmol/L silvestrol during the experiment. After differentiation, cells were washed with Seahorse XF RPMI medium pH 7.4 (Agilent), incubated for 60 minutes at 37°C, and OCR and ECAR were measured for a total period of 160 minutes in the absence of silvestrol. Cells were stimulated after 30 minutes. Monocytes were stimulated with 100 ng/mL lipopolysaccharides (LPS) and 20 ng/mL IFN‐γ, macrophages were stimulated with 20 ng/mL IFN‐γ while dendritic cells were stimulated with a Stimulation‐Mix containing 5 ng/mL of human TNF‐α, IL‐6, IL‐1β and 500 ng/mL PGE_2_. Cells were measured as octuplicates (3 × 10^4^ cells per well) using the Seahorse XFe96 Analyzer (Agilent) and analysed by Wave Software (Agilent).

### 2.10 Statistics

Results are presented as means ± standard errors (SEM). For all calculations and creation of graphs, GraphPad Prism 8 was used and *P* < .05 was considered as the threshold for significance. Applied statistical analysis is denoted in the figure legends. In every test, silvestrol treatment was compared to vehicle.

## 3 RESULTS

### 3.1 Silvestrol and cell viability

**FIGURE 1:**
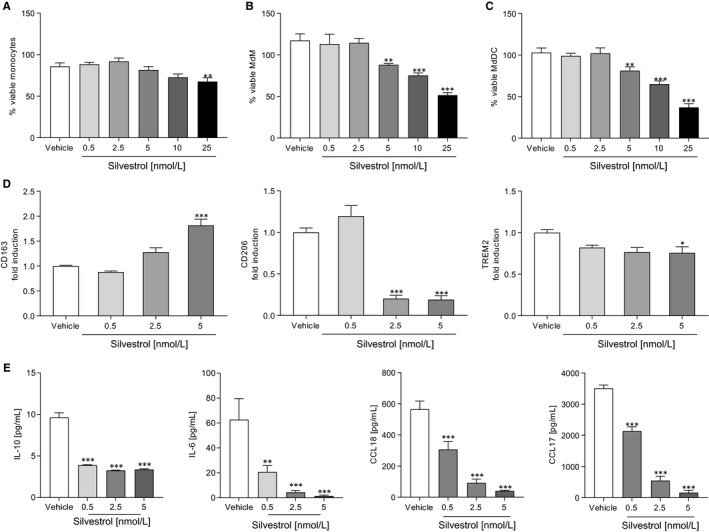
Effects of silvestrol on immune cell viability and macrophage differentiation. A‐C, Percentage of viable human monocytes (A), MdMs (B) and MdDCs (C) in the presence or absence of different concentrations of silvestrol as indicated or vehicle (DMSO) was determined by Orangu assay in triplicates. Human monocytes were isolated from buffy coats, stimulated and incubated for 48 h (monocytes), 5 d (MdDCs) or 7 d (MdMs). MdM medium was completely renewed after 3 d. Monocytes were incubated without further stimulation while MdMs and MdDCs were stimulated with GM‐CSF (10 ng/mL) or GM‐CSF (10 ng/mL) and IL‐4 (10 ng/mL), respectively. Percentage values were calculated with cells cultivated only in medium as reference (n = 4). D and E, Human macrophages were differentiated as described above in the presence of different concentrations of silvestrol as indicated or vehicle (DMSO) for 7 d. D, Surface marker expression of MdMs was measured by MACSQuant^®^ Analyzer 10 in triplicates. Fold induction of the geometric mean of the fluorescence intensity was calculated by referring treated cells to vehicle controls (n = 10). E, Released cytokines in the supernatant of MdMs after differentiation in the presence of different concentrations of silvestrol or vehicle (DMSO) for 7 d. Cytokine concentrations were measured by cytometric bead array or ELISA in triplicates (n = 6). For statistical analysis, one‐way ANOVA with Dunnett's multiple comparisons test (A‐E) was used. *P < .05, **P < .01, ***P < .001 indicate significant difference between silvestrol‐ and vehicle‐treated samples

Since viable cells are a prerequisite for further experiments, the influence of silvestrol on cell viability was examined first. Using the Orangu assay, the percentage of viable cells after silvestrol or vehicle (DMSO) treatment was determined in comparison to cells cultivated in pure medium. A concentration of 5 nmol/L silvestrol reduced, by about 20%, cell viability of monocytes, MdDCs and MdMs (Figure 1A‐C). Silvestrol concentrations of 10 and 25 nmol/L significantly reduced the viability of MdMs and MdDCs (Figure 1B,C). Therefore, for further investigations, silvestrol was used at a maximum concentration of 5 nmol/L.

### 3.2 Silvestrol modifies differentiation of MdM

To investigate whether silvestrol influences the differentiation of monocytes to macrophages, monocytes were differentiated to MdMs by addition of GM‐CSF. To characterize the inflammatory status of the cells, surface markers that are up‐regulated during differentiation (CD80, CD86, CD163, CD206, TREM2, HLA‐DR) and cytokines that are released during differentiation (IL‐10, IL‐6, CCL18, CCL17)^25^ were determined. CD163, an immune sensor for bacteria, was significantly increased at 5 nmol/L silvestrol, whereas the pattern recognition receptor CD206 and TREM2 were significantly reduced (Figure 1D). Pro‐inflammatory surface markers CD80, CD86 and HLA‐DR were not significantly altered at the silvestrol concentrations used (Figure S1A). Interestingly, silvestrol significantly reduced the release of the anti‐inflammatory cytokine IL‐10, the pro‐inflammatory cytokine IL‐6 and the chemokines CCL17 and CCL18 even at concentrations of 0.5 nmol/L (Figure 1E). These data indicate that silvestrol alters the release of cytokines/chemokines and the expression of surface markers in differentiating macrophages, without generation of a recognized macrophage phenotype.

### 3.3 Silvestrol reduces release of chemotaxins but promotes inflammatory markers in M1 MdMs

**FIGURE 2:**
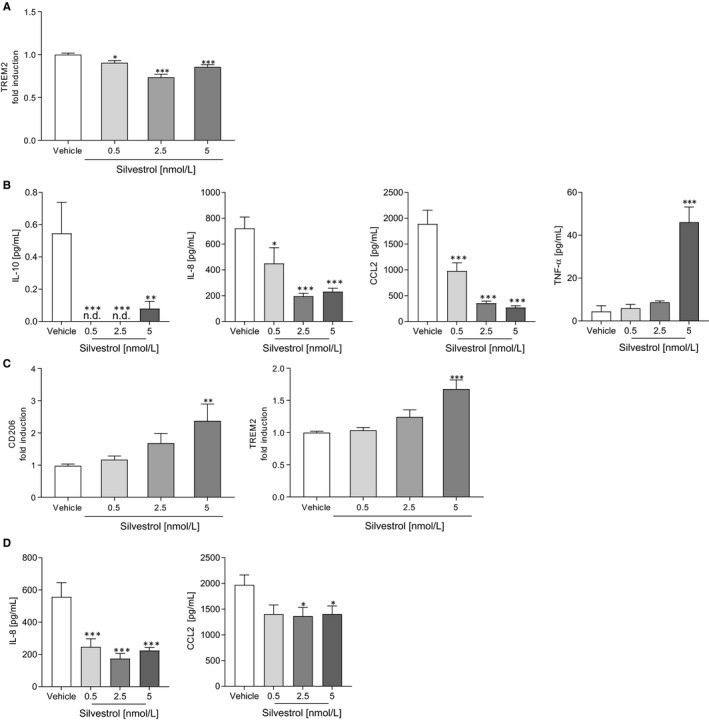
Influence of silvestrol on M1 and M2 polarized macrophage surface marker and cytokines. Human monocytes were isolated from buffy coats and differentiated to M1 or M2 MdMs for 7 d using GM‐CSF (10 ng/mL) or M‐CSF (50 ng/mL), respectively. After differentiation, cells were polarized with 20 ng/mL IFN‐γ (M1 MdMs) or 10 ng/mL IL‐4 (M2 MdMs) in the presence of different concentrations of silvestrol as indicated or vehicle (DMSO) for 48 h or 24 h, respectively. A and C, Surface marker expression of M1 (A) or M2 (C) MdMs was measured by MACSQuant^®^ Analyzer 10 in triplicates. Fold induction of the geometric mean of the fluorescence intensity was calculated by referring treated cells to vehicle controls (n = 10). B and D, Released cytokines in the supernatant of M1 (B) or M2 (D) MdMs with 0.5, 2.5 or 5 nmol/L silvestrol or vehicle (DMSO) treatment. Cytokine concentrations were measured by cytometric bead array or ELISA in triplicates (n = 6). For statistical analysis, one‐way ANOVA with Dunnett's multiple comparisons test (A‐D) was used. *P < .05, **P < .01, ***P < .001 indicate significant difference between silvestrol‐ and vehicle‐treated samples

Next, we investigated whether silvestrol influences the polarization of MdMs. For this, MdMs were polarized with IFN‐γ to M1 MdMs. Surface markers (CD80, CD86, CD163, CD206, TREM2, HLA‐DR) that are up‐regulated and cytokines/chemokines (IL‐23, IL‐10, IL‐8, TNF‐α, CCL18, CXCL10, CCL2) released during M1‐polarization^25^ were determined. In M1 MdMs, silvestrol only slightly reduced the expression of the surface marker TREM2 (Figure 2A). The surface markers CD80, CD86, CD163, CD206 and HLA‐DR were not significantly changed at 5 nmol/L silvestrol (Figure S2A). However, silvestrol significantly reduced the release of IL‐10, and the chemotactic chemokines IL‐8 and CCL2, whereas TNF‐α was significantly increased in M1 MdMs (Figure 2B). The release of CXCL10 and IL‐23 was not influenced by silvestrol (Figure 2B). These data indicate that silvestrol could potentially impair the recruitment of further immune cells as a result of the reduction of IL‐8 and CCL2 release, whereas it might promote a pro‐inflammatory environment by increasing TNF‐α and decreasing IL‐10 release.

### 3.4 Silvestrol promotes anti‐inflammatory markers in M2 MdM

MdMs were polarized by IL‐4 to M2 MdMs and surface markers (CD80, CD86, CD163, CD206, TREM2, HLA‐DR) that are up‐regulated and cytokines/chemokines (IL‐23, IL‐10, IL‐8, TNF‐α, CCL18, CXCL10, CCL2) released during M2‐polarization^25^ were determined. In M2 MdMs, expression of the M2‐specific surface markers CD206 and TREM2 was significantly increased by 5 nmol/L silvestrol (Figure 2C), whereas the release of chemotaxins IL‐8 and CCL2 was significantly reduced by silvestrol (Figure 2D). Surface markers CD80, CD86, CD163 and HLA‐DR (Figure S3A) and the release of IL‐23, IL‐10, TNF‐α and CXCL10 were not significantly influenced by silvestrol (Figure S3B). These data indicate that silvestrol promotes the anti‐inflammatory potential of M2 macrophages, reducing the release of chemokines that may recruit other immune cells and promoting the expression of M2 phenotype surface markers, thus suggesting the potential to facilitate inflammation resolution.

### 3.5 Silvestrol influences the differentiation of monocytes to MdDCs

**FIGURE 3:**
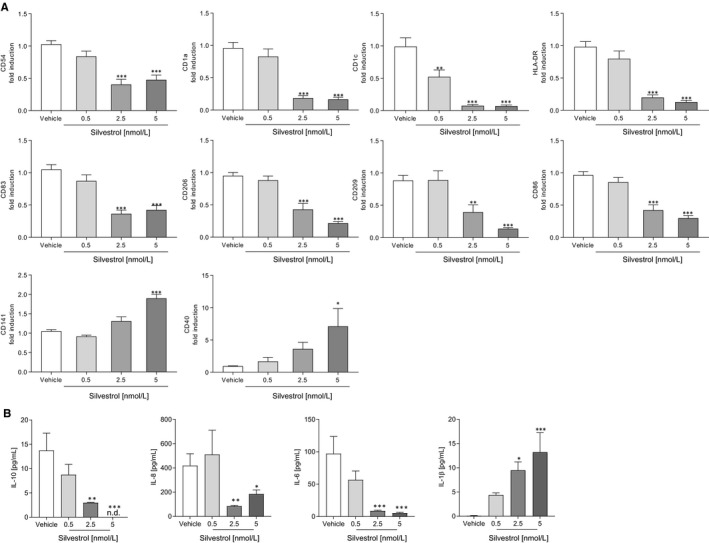
Effects of silvestrol on surface markers and cytokine release of MdDCs. Human monocytes were differentiated to MdDCs for 5 d with GM‐CSF (10 ng/mL) and IL‐4 (10 ng/mL) in the presence or absence of different concentrations of silvestrol as indicated or vehicle (DMSO). A, Surface marker expression was measured by MACSQuant^®^ Analyzer 10 in triplicates. Fold induction of the geometric mean of the fluorescence intensity was calculated by referring treated cells to vehicle controls (n = 6). B, Released cytokines in the supernatant were measured by cytometric bead array in triplicates (n = 6). For statistical analysis, one‐way ANOVA with Dunnett's multiple comparisons test (A‐B) was used. *P < .05, **P < .01, ***P < .001 indicate significant difference between silvestrol‐ and vehicle‐treated samples

Because not only macrophages but also dendritic cells are important players in the immune response to pathogens, the effects of silvestrol on dendritic cell differentiation were analysed. For this purpose, monocytes were differentiated to MdDCs by the addition of GM‐CSF and IL‐4 over 5 days. Surface markers (CD1a, CD1c, CD40, CD54, CD80, CD83, CD86, CD141, CD197, CD206, CD209, HLA‐DR) that are up‐regulated and cytokines/chemokines (IL‐23, IL‐12, IL‐10, IL‐8, IL‐6, IL‐1β) released during dendritic cell differentiation^26^ were determined. Several surface markers of MdDCs were down‐regulated after differentiation in the presence of silvestrol. CD54, CD1a, CD1c, HLA‐DR, CD83, CD206, CD209 and CD86 were all significantly reduced at 2.5 and 5 nmol/L silvestrol. Only the expression of CD141 and CD40 was significantly increased at 5 nmol/L silvestrol (Figure 3A). The surface markers CD80 and CD197 were not modified (Figure S4A).

Silvestrol led to a concentration‐dependent reduction of IL‐10, IL‐8 and IL‐6 release. IL‐1β release was significantly increased after differentiation of MdDCs by silvestrol (Figure 3B). IL‐12 was not detectable either in vehicle or in silvestrol‐treated MdDCs, and IL‐23 release was not affected (Figure S4B). These data indicate that silvestrol has a predominantly inhibitory effect on markers of the differentiation status of dendritic cells, but enhances IL‐1ß release as well as CD141 and CD40 expression, at least partially reflecting potential promotion of cytotoxic T‐cell responses, for instance to combat viral infections.^27^, ^28^

### 3.6 Silvestrol influences the activation of dendritic cells

**FIGURE 4:**
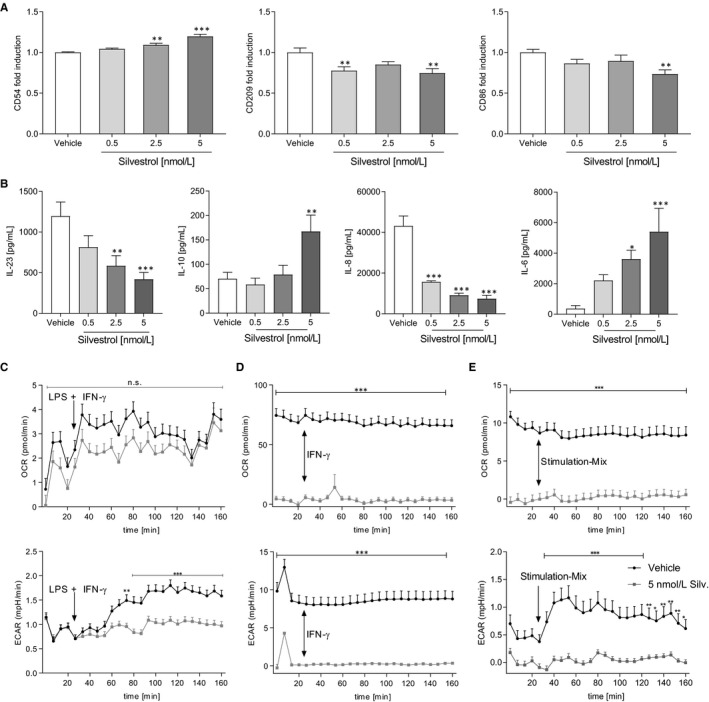
Effects of silvestrol on activated MdDCs and energy metabolism. A and B, MdDCs were initially differentiated without silvestrol and further activated with a mixture of 5 ng/mL IL‐1β, 5 ng/mL IL‐6, 5 ng/mL TNF‐α, 500 ng/mL PGE_2_ for 1 d in the presence of different concentrations of silvestrol as indicated or vehicle (DMSO). A, Surface marker expression was measured by MACSQuant^®^ Analyzer 10 in triplicates. Fold induction of the geometric mean of the fluorescence intensity was calculated by referring treated cells to vehicle controls (n = 6). B, Released cytokines in the supernatant were measured by cytometric bead array or ELISA (n = 6). C‐E, Human monocytes were isolated from buffy coats and incubated for 48 h (monocytes, C), 7 d (MdMs, D) or 5 d (MDDCs, E). MdM medium was completely renewed after 3 d. Monocytes were incubated without further stimulation while MdMs and MdDCs were stimulated with GM‐CSF (10 ng/mL) or GM‐CSF (10 ng/mL) and IL‐4 (10 ng/mL), respectively. During differentiation, cells were cultivated in the presence of 5 nmol/L silvestrol. After differentiation, oxygen consumption rate (OCR) and extracellular acidification rate (ECAR) were measured with the Seahorse XFe96 analyzer (Agilent) over a total time period of 160 min in the absence of silvestrol. Stimulation with LPS + IFN‐γ (C, c [LPS] = 100 ng/mL, c [IFN‐γ] = 20 ng/mL), IFN‐γ [D, c (IFN‐γ] = 20 ng/mL) or a Stimulation‐Mix containing PGE2, IL‐1β, IL‐6 and TNF‐α (E, c [PGE2] = 500 ng/mL, c [IL‐1β, IL‐6, TNF‐α] = 5 ng/ml) started after 30 min (n = 3). For statistical analysis, one‐way ANOVA with Dunnett's multiple comparisons test (A‐B) or two‐way ANOVA with Bonferroni's multiple comparisons test (C‐E) were used. *P < .05, **P < .01, ***P < .001 show significant difference between silvestrol and vehicle treatment

Next, we investigated whether silvestrol influences the activation of dendritic cells. Therefore, MdDCs were activated by IL‐1ß, TNF‐α, IL‐6 and PGE_2_. Interestingly, the expression of CD54 was moderately up‐regulated while CD209 and CD86 were significantly down‐regulated by 5 nmol/L silvestrol (Figure 4A). The expression of antigen‐presenting markers (CD1a, CD1c, HLA‐DR), co‐stimulation markers (CD40, CD80), activation markers (CD197, C83), a phagocytosis marker (CD206) and CD141 was not altered (Figure S5). Furthermore, silvestrol reduced concentration dependent the release of IL‐23 and IL‐8 and increased concentration dependent the release of IL‐10 and IL‐6 (Figure 4B) which could lead to a modified T helper cell phenotype in vivo.

### 3.7 Silvestrol reduces energy metabolism

Because silvestrol had considerable impact on macrophages and dendritic cells, we investigated whether this might be a result of modified energy metabolism. Therefore, ECAR, a marker for glycolysis, and OCR, a marker for mitochondrial respiration, were determined (Figure 4C‐E). In monocytes, 5 nmol/L silvestrol failed to show significant effects on OCR while ECAR in silvestrol‐treated cells could not be induced by stimulation with LPS and IFN‐γ. In MdMs, OCR and ECAR were both significantly reduced by silvestrol treatment. Stimulation with IFN‐γ did not influence the energy metabolism. OCR in MdDCs was significantly reduced by silvestrol and could not be stimulated with a Stimulation‐Mix. However, ECAR in vehicle‐treated cell was susceptible to stimulation, while silvestrol‐treated cells were not influenced by stimulation. These data indicate that silvestrol suppresses the energy metabolism of immune cells by impairing glycolysis and mitochondrial respiration.

## 4 DISCUSSION

**FIGURE 5:**
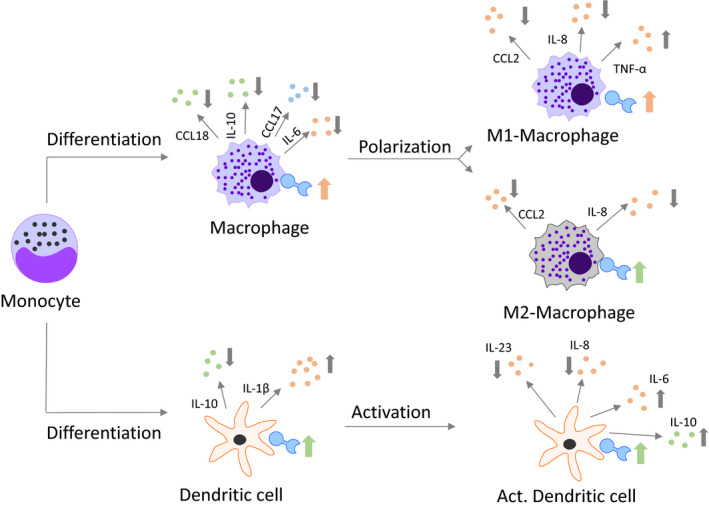
Overview of silvestrol‐mediated effects on MdMs and MdDCs. Effects of 5 nmol/L silvestrol during the differentiation of monocytes to MdMs and MdDCs, during the polarization of MdMs to M1 or M2 MdMs and during the activation of MdDCs are shown. Only changes of at least 20% are included in this overview. Predominantly pro‐inflammatory cytokines/chemokines are labelled in bright red, predominantly anti‐inflammatory cytokines/chemokines are shown in bright‐green and cytokines/chemokines with pro‐ and anti‐inflammatory potential are shown in bright blue. The direction of the arrows indicates a decrease (↓) or an increase (↑). For greater clarity, because the regulation of the surface marker was not as pronounced as the cytokine/chemokine alterations, the changes in the surface markers are shown as tendencies. Thus, a pink arrow indicates that the majority of the surface marker reflect a pro‐inflammatory phenotype, a green arrow demonstrates that the majority of the surface marker reflect anti‐inflammatory characteristics and both arrows indicate that the regulation tended towards equilibrium

We have analysed the effects of the antiviral compound and specific eIF4A inhibitor silvestrol on immune cell differentiation and activation. Silvestrol exerted a pronounced influence on the inflammatory status of immune cells in vitro in a variety of ways (summarized in Figure 5). The effects observed depended on the immune cell types and their stages of activation. This raises the question as to how silvestrol mediates these different effects. Silvestrol is known to inhibit elF4A, a crucial component of the eukaryotic translation initiation complex 4F (eIF4F). This complex binds to the 5′ methyl guanosine cap structure of mRNA and recruits the small ribosomal subunit (40S) to mRNAs to initiate cap‐dependent translation. ElF4A is an RNA helicase which prepares the mRNA for translation. Wolfe et al demonstrated that the inhibition of elF4A by silvestrol reduces the translation of only a minor set of selected mRNAs in cancer cell lines.^29^ However, the effect of elF4A inhibition by silvestrol in immune cells has not been extensively investigated until now and our data indicate a complex pattern of effects.

During differentiation of macrophages, silvestrol may impair this process, suppressing the appearance of an anti‐inflammatory phenotype, with reduced pathogen recognition, and modify the recruitment of regulatory immune cells. CD206, which is down‐regulated by silvestrol, is a pattern recognition receptor that recognizes microbial carbohydrates on pathogens such as bacteria and viruses and mediates their phagocytosis.^30^ This could potentially impair the recognition and the phagocytosis of pathogens by macrophages. However, CD163, an immune sensor for bacteria,^31^ is up‐regulated by silvestrol treatment. IL‐6 promotes differentiation of M2 macrophages.^32^ Therefore, the silvestrol‐induced reduction of IL‐6 would facilitate pro‐inflammatory effects. Silvestrol may also reduce the recruitment of regulatory T cells by reducing release of CCL17 and CCL18. The reduced CCL18 could be a consequence of the reduced IL‐10 expression, because IL‐10 induces the release of CCL18 in antigen‐presenting cells.^33^ Taken together, silvestrol seems to impair the differentiation of macrophages to a phenotype that recruits anti‐inflammatory immune cells and recognizes pathogens.

The pro‐inflammatory potential of M1 MdMs appears to be enhanced by the increased release of the pro‐inflammatory cytokine TNF‐α, but the attraction of further immune cells by these cells is impaired by reduced release of chemokines (IL‐8, CCL2). During M1‐polarization, silvestrol increases TNF‐α and decreases IL‐8 as well as CCL2 release. The attenuated release of IL‐8 and CCL2 could result in reduced recruitment of neutrophils and monocytes and finally to attenuation of the immune response. On the other hand, increased TNF‐α release could strengthen the inflammatory response because TNF‐α further activates macrophages. IFN‐γ, which is used to generate M1 macrophages in vitro, mediates its effect among others via STAT‐1 and nuclear factor 'kappa‐light‐chain‐enhancer' of activated B cells (NFκB) signalling. In osteoblasts, it has been shown that down‐regulation of STAT‐1 or STAT‐3 reduces the expression of CCL‐2^34^ and in a lung cancer cell line, STAT‐1 was linked to IL‐8 expression,^35^ whereas TNF‐α synthesis is regulated via NFκB signalling. Bearing in mind that silvestrol reduces STAT‐1 and STAT‐3 expression via eIF4A inhibition,^17^, ^36^ it seems possible that its inhibition of IL‐8 and CCL2 release is a result of reduced STAT‐1/3 expression.

Silvestrol potentially amplifies the anti‐inflammatory potential of M2 MdMs, diminishing release of chemokines which recruit neutrophils and monocytes. In contrast to macrophage differentiation, silvestrol induces the expression of CD206. Because CD206 induces phagocytosis, silvestrol could lead to a more effective removal of bacterial and viral particles. Furthermore, silvestrol led to a reduction of IL‐8 and CCL2, which would tend to reduce recruitment of neutrophils and monocytes. Consequently, silvestrol might promote the initiation of inflammatory resolution by M2 macrophages, an effect likely to be strengthened by the reduction of pro‐inflammatory markers during macrophage differentiation. The lack of effect of silvestrol on release of the anti‐inflammatory IL‐10 under these conditions is unexpected. However, we have shown in previous investigations of drug effects on macrophage polarization that pharmacological agents can mediate macrophage phenotype changes without modifying all the expected markers in the same way.^25^, ^37^ This is not surprising on the basis of their different mechanisms of action.

In dendritic cells, silvestrol seems to suppress maturation because most of the differentiation markers and several cytokines were down‐regulated. The differentiation markers such as CD83, HLA‐DR and CD86 were reduced, and fewer amounts of cytokines such as IL‐6, IL‐8 and IL‐10 were released. Inhibition of DC differentiation might also be a result of effects on STAT‐3 signalling. Numerous reports indicate that STAT‐3 suppresses DC maturation and activation by down‐regulating the expression of MHC class II and co‐stimulatory molecules on DCs and STAT‐3‐mediated inhibition of toll‐like receptor (TLR)‐induced pro‐inflammatory mediators.^38^ Repression of DC maturation/function can also be achieved via IL‐6‐STAT‐3‐ or IL‐10‐STAT‐3‐mediated signalling directly or indirectly through inhibitory molecules that induce IL‐6.^39^, ^40^ IL‐6 and IL‐10 were both up‐regulated by silvestrol during dendritic cell activation, which might explain the suppressive effects of silvestrol. However, the fact remains that during dendritic cell differentiation, IL‐6 and IL‐10 release were both reduced by silvestrol. Additionally, it has been shown that silvestrol also inhibits phosphorylation of STAT‐3, which would lead to opposite effects.^36^ Therefore, another signalling pathway might be involved. In tumour models, silvestrol reduced STAT‐1 mRNA.^17^, ^36^ Because STAT‐1 is essential for DC differentiation, inhibition by silvestrol might explain the observed effects on reduced dendritic cell differentiation and activation.^41^, ^42^

Silvestrol may also modify immune cell infiltration by reducing release of chemokines and by down‐regulation of adhesion molecules. CD54, a surface protein of the integrin family, was reduced by silvestrol during dendritic cell differentiation. This is in line with other antipathogenic drugs such as macrolides that also decrease the expression of CD54^43^, ^44^ and inhibit neutrophil migration.^45^ Moreover, silvestrol reduced the release of chemotactic CCL18 and CCL17 during macrophage differentiation, of CXCL8 and CCL2 in M1 and M2 macrophages and of CXCL8 during dendritic cell differentiation and activation. Similarly, tetracyclines down‐regulate the production of LPS‐induced chemokines, such as CXCL8, CCL3 and CCL4 in THP‐1 cells via NFκB signalling pathways.^46^ The antiviral drug acyclovir inhibits the migratory potential of breast cancer cells.^47^ These data indicate that silvestrol possibly attenuates inflammation by a reduction of immune cell attraction to the lesion site.

Our data further reveal that silvestrol impairs energy metabolism in myeloid cells. In this respect, it is noteworthy that silvestrol inhibits the proviral integration site for moloney murine leukaemia virus (PIM)1 and PIM2—two kinases involved among others in mechanisms of energy metabolism.^48^ PIM inhibition via the mechanistic target of rapamycin complex 1 (mTORC1) pathway leads to reduced glycolysis in mouse embryonic fibroblasts.^49^, ^50^ Therefore, silvestrol possibly inhibits glycolysis via the inhibition of PIM kinases in MdMs and MdDCs. Furthermore, the effect of silvestrol on energy metabolism and on the immune function of myeloid cells raises the question as to whether these are independent or interdependent effects. Anti‐inflammatory immune cells such as M2 macrophages use predominantly oxidative phosphorylation, whereas pro‐inflammatory cells such as M1 macrophages select glycolysis as their main energy source.^51^ Silvestrol amplifies the anti‐inflammatory phenotype of M2 macrophages, but it did not amplify their oxidative phosphorylation. Consequently, the effects of silvestrol on the inflammatory status of myeloid cells are probably independent of its effect on energy metabolism.

Our findings indicate that depending on the immune cell type and on the differentiation/activation status of the cells, silvestrol mediates differing effects. At the beginning of an infection (differentiation of monocytes to macrophages or dendritic cells), silvestrol could potentially suppress the generation of anti‐inflammatory phenotypes of macrophages and dendritic cells and support host defence against pathogens. On the other hand, during pathogen‐induced inflammation, silvestrol seems to accelerate the transition from pro‐ to anti‐inflammatory status, reducing bystander tissue injury and promoting inflammation resolution. In view of the various pro‐ and anti‐inflammatory effects of silvestrol on immune cells, further studies are needed to assess whether silvestrol is able to assist immune defence against pathogens at the beginning of an infection and promote the resolution of inflammation as infection declines.

## CONFLICT OF INTEREST

The authors declare no commercial or financial conflict of interest.

## AUTHOR CONTRIBUTIONS

L.B investigated, involved in formal analysis and data curation, and wrote original draft and revised. G.G conceptualized. M. J. P., A. G and S. S. conceptualized and wrote original draft and revised.

## Supporting information

Fig S1

Fig S2

Fig S3

Fig S4

Fig S5

## Data Availability

The data that support the findings of this study are available from the corresponding author upon reasonable request.

## References

[1] WHO Ebola Responses Team , Agua‐Agum J , Ariyarajah A , et al. West African Ebola epidemic after one year–slowing but not yet under control. N Engl J Med. 2015;372:584‐587. 25539446 10.1056/NEJMc1414992 PMC4368109

[2] Kim S , Hwang BY , Su BN , et al. Silvestrol, a potential anticancer rocaglate derivative from Aglaia foveolata, induces apoptosis in LNCaP cells through the mitochondrial/apoptosome pathway without activation of executioner caspase‐3 or ‐7. Anticancer Res. 2007;27:2175‐2183. 17695501 PMC2787233

[3] Lucas DM , Edwards RB , Lozanski G , et al. The novel plant‐derived agent silvestrol has B‐cell selective activity in chronic lymphocytic leukemia and acute lymphoblastic leukemia in vitro and in vivo. Blood. 2009;113:4656‐4666. 19190247 10.1182/blood-2008-09-175430 PMC2680369

[4] Kogure T , Kinghorn AD , Yan I , et al. Therapeutic potential of the translation inhibitor silvestrol in hepatocellular cancer. PLoS ONE. 2013;8:e76136. 24086701 10.1371/journal.pone.0076136 PMC3784426

[5] Chen WL , Pan L , Kinghorn AD , Swanson SM , Burdette JE . Silvestrol induces early autophagy and apoptosis in human melanoma cells. BMC Cancer. 2016;16:17. 26762417 10.1186/s12885-015-1988-0 PMC4712514

[6] Bordeleau ME , Robert F , Gerard B , et al. Therapeutic suppression of translation initiation modulates chemosensitivity in a mouse lymphoma model. J Clin Invest. 2008;118:2651‐2660. 18551192 10.1172/JCI34753 PMC2423864

[7] Sadlish H , Galicia‐Vazquez G , Paris CG , et al. Evidence for a functionally relevant rocaglamide binding site on the eIF4A‐RNA complex. ACS Chem Biol. 2013;8:1519‐1527. 23614532 10.1021/cb400158t PMC3796129

[8] Muller KH , Kakkola L , Nagaraj AS , Cheltsov AV , Anastasina M , Kainov DE . Emerging cellular targets for influenza antiviral agents. Trends Pharmacol Sci. 2012;33:89‐99. 22196854 10.1016/j.tips.2011.10.004

[9] Gerold G , Pietschmann T . Opportunities and risks of host‐targeting antiviral strategies for hepatitis C. Curr Hepat Rep. 2013;12:200‐213. 32214912 10.1007/s11901-013-0187-1 PMC7089091

[10] Muller C , Schulte FW , Lange‐Grunweller K , et al. Broad‐spectrum antiviral activity of the eIF4A inhibitor silvestrol against corona‐ and picornaviruses. Antiviral Res. 2018;150:123‐129. 29258862 10.1016/j.antiviral.2017.12.010 PMC7113723

[11] Biedenkopf N , Lange‐Grunweller K , Schulte FW , et al. The natural compound silvestrol is a potent inhibitor of Ebola virus replication. Antiviral Res. 2017;137:76‐81. 27864075 10.1016/j.antiviral.2016.11.011

[12] Elgner F , Sabino C , Basic M , Ploen D , Grunweller A , Hildt E . Inhibition of zika virus replication by silvestrol. Viruses. 2018;10:E149. 29584632 10.3390/v10040149 PMC5923443

[13] Henss L , Scholz T , Grunweller A , Schnierle BS . Silvestrol inhibits chikungunya virus replication. Viruses. 2018;10:E592. 30380742 10.3390/v10110592 PMC6266838

[14] Glitscher M , Himmelsbach K , Woytinek K , et al. Inhibition of hepatitis E virus spread by the natural compound silvestrol. Viruses. 2018;10(6):E301. 29865243 10.3390/v10060301 PMC6024817

[15] Muraille E , Leo O , Moser M . TH1/TH2 paradigm extended: macrophage polarization as an unappreciated pathogen‐driven escape mechanism? Front Immunol. 2014;5:603. 25505468 10.3389/fimmu.2014.00603 PMC4244692

[16] Blum L , Schiffmann S , Parnham M . Immunomodulation by antibiotics In: Budimir A, ed. Fighting Antimicrobial Resistance. Zagreb, Croatia: IAPC Publishing, 2018.

[17] Cerezo M , Guemiri R , Druillennec S , et al. Translational control of tumor immune escape via the eIF4F‐STAT1‐PD‐L1 axis in melanoma. Nat Med. 2018;24:1877‐1886. 30374200 10.1038/s41591-018-0217-1

[18] Regis G , Pensa S , Boselli D , Novelli F , Poli V . Ups and downs: the STAT1:STAT3 seesaw of Interferon and gp130 receptor signalling. Semin Cell Dev Biol. 2008;19:351‐359. 18620071 10.1016/j.semcdb.2008.06.004

[19] Atri C , Guerfali FZ , Laouini D . Role of human macrophage polarization in inflammation during infectious diseases. Int J Mol Sci. 2018;19:1801‐1816. 10.3390/ijms19061801 PMC6032107 29921749

[20] Gazi U , Martinez‐Pomares L . Influence of the mannose receptor in host immune responses. Immunobiology. 2009;214:554‐561. 19162368 10.1016/j.imbio.2008.11.004

[21] Mason CP , Tarr AW . Human lectins and their roles in viral infections. Molecules. 2015;20:2229‐2271. 25642836 10.3390/molecules20022229 PMC6272597

[22] Savill J . Apoptosis in resolution of inflammation. Kidney Blood Press Res. 2000;23:173‐174. 11031712

[23] Solari R , Pease JE . Targeting chemokine receptors in disease–a case study of CCR4. Eur J Pharmacol. 2015;763:169‐177. 25981299 10.1016/j.ejphar.2015.05.018 PMC4784718

[24] Chenivesse C , Tsicopoulos A . CCL18 ‐ Beyond chemotaxis. Cytokine. 2018;109:52‐56. 29402725 10.1016/j.cyto.2018.01.023

[25] Shiratori H , Feinweber C , Luckhardt S , et al. An in vitro test system for compounds that modulate human inflammatory macrophage polarization. Eur J Pharmacol. 2018;833:328‐338. 29920284 10.1016/j.ejphar.2018.06.017

[26] Collin M , McGovern N , Haniffa M . Human dendritic cell subsets. Immunology. 2013;140:22‐30. 23621371 10.1111/imm.12117 PMC3809702

[27] Meixlsperger S , Leung CS , Ramer PC , et al. CD141+ dendritic cells produce prominent amounts of IFN‐alpha after dsRNA recognition and can be targeted via DEC‐205 in humanized mice. Blood. 2013;121:5034‐5044. 23482932 10.1182/blood-2012-12-473413 PMC3689250

[28] Yu CI , Becker C , Metang P , et al. Human CD141+ dendritic cells induce CD4+ T cells to produce type 2 cytokines. J Immunol. 2014;193:4335‐4343. 25246496 10.4049/jimmunol.1401159 PMC4201960

[29] Wolfe AL , Singh K , Zhong Y , et al. RNA G‐quadruplexes cause eIF4A‐dependent oncogene translation in cancer. Nature. 2014;513:65‐70. 25079319 10.1038/nature13485 PMC4492470

[30] Azad AK , Rajaram MVS , Schlesinger LS . Exploitation of the Macrophage Mannose Receptor (CD206) in infectious disease diagnostics and therapeutics. J Cytol Mol Biol. 2014;1(1). 10.13188/2325-4653.1000003 PMC3963702 24672807

[31] Fabriek BO , van Bruggen R , Deng DM , et al. The macrophage scavenger receptor CD163 functions as an innate immune sensor for bacteria. Blood. 2009;113:887‐892. 18849484 10.1182/blood-2008-07-167064

[32] Chomarat P , Banchereau J , Davoust J , Palucka AK . IL‐6 switches the differentiation of monocytes from dendritic cells to macrophages. Nat Immunol. 2000;1:510‐514. 11101873 10.1038/82763

[33] van der Voort R , Kramer M , Lindhout E , et al. Novel monoclonal antibodies detect elevated levels of the chemokine CCL18/DC‐CK1 in serum and body fluids in pathological conditions. J Leukoc Biol. 2005;77:739‐747. 15713699 10.1189/jlb.0804435

[34] Kok SH , Hong CY , Kuo MY , et al. Oncostatin M‐induced CCL2 transcription in osteoblastic cells is mediated by multiple levels of STAT‐1 and STAT‐3 signaling: an implication for the pathogenesis of arthritis. Arthritis Rheum. 2009;60:1451‐1462. 19404962 10.1002/art.24452

[35] Huang Q , Duan I , Qian X , et al. Corrigendum: IL‐17 promotes angiogenic factors IL‐6, IL‐8, and Vegf production via Stat1 in lung adenocarcinoma. Sci Rep. 2017;7:39566. 28074916 10.1038/srep39566 PMC5225604

[36] Patton JT , Lustberg ME , Lozanski G , et al. The translation inhibitor silvestrol exhibits direct anti‐tumor activity while preserving innate and adaptive immunity against EBV‐driven lymphoproliferative disease. Oncotarget. 2015;6:2693‐2708. 25393910 10.18632/oncotarget.2098 PMC4413611

[37] Vrancic M , Banjanac M , Nujic K , et al. Azithromycin distinctively modulates classical activation of human monocytes in vitro. Br J Pharmacol. 2012;165:1348‐1360. 21726210 10.1111/j.1476-5381.2011.01576.x PMC3372721

[38] Hillmer EJ , Zhang H , Li HS , Watowich SS . STAT3 signaling in immunity. Cytokine Growth Factor Rev. 2016;31:1‐15. 27185365 10.1016/j.cytogfr.2016.05.001 PMC5050093

[39] Corinti S , Albanesi C , la Sala A , Pastore S , Girolomoni G . Regulatory activity of autocrine IL‐10 on dendritic cell functions. J Immunol. 2001;166:4312‐4318. 11254683 10.4049/jimmunol.166.7.4312

[40] Liang S , Ristich V , Arase H , Dausset J , Carosella ED , Horuzsko A . Modulation of dendritic cell differentiation by HLA‐G and ILT4 requires the IL‐6–STAT3 signaling pathway. Proc Natl Acad Sci USA. 2008;105:8357‐8362. 18550825 10.1073/pnas.0803341105 PMC2448841

[41] McCormick SM , Heller NM . Regulation of macrophage, dendritic cell, and microglial phenotype and function by the SOCS proteins. Front Immunol. 2015;6:549. 26579124 10.3389/fimmu.2015.00549 PMC4621458

[42] Jackson SH , Yu CR , Mahdi RM , Ebong S , Egwuagu CE . Dendritic cell maturation requires STAT1 and is under feedback regulation by suppressors of cytokine signaling. J Immunol. 2004;172:2307‐2315. 14764699 10.4049/jimmunol.172.4.2307

[43] Khair OA , Devalia JL , Abdelaziz MM , Sapsford RJ , Davies RJ . Effect of erythromycin on Haemophilus influenzae endotoxin‐induced release of IL‐6, IL‐8 and sICAM‐1 by cultured human bronchial epithelial cells. Eur Respir J. 1995;8:1451‐1457. 8575568

[44] Kawasaki S , Takizawa H , Ohtoshi T , et al. Roxithromycin inhibits cytokine production by and neutrophil attachment to human bronchial epithelial cells in vitro. Antimicrob Agents Chemother. 1998;42:1499‐1502. 9624502 10.1128/aac.42.6.1499 PMC105630

[45] Oda H , Kadota J , Kohno S , Hara K . Erythromycin inhibits neutrophil chemotaxis in bronchoalveoli of diffuse panbronchiolitis. Chest. 1994;106:1116‐1123. 7924482 10.1378/chest.106.4.1116

[46] Sun J , Shigemi H , Tanaka Y , Yamauchi T , Ueda T , Iwasaki H . Tetracyclines downregulate the production of LPS‐induced cytokines and chemokines in THP‐1 cells via ERK, p38, and nuclear factor‐kappaB signaling pathways. Biochem Biophys Rep. 2015;4:397‐404. 29124230 10.1016/j.bbrep.2015.11.003 PMC5669446

[47] Shaimerdenova M , Karapina O , Mektepbayeva D , Alibek K , Akilbekova D . The effects of antiviral treatment on breast cancer cell line. Infect Agent Cancer. 2017;12:18. 28344640 10.1186/s13027-017-0128-7 PMC5364572

[48] Schatz JH , Oricchio E , Wolfe AL , et al. Targeting cap‐dependent translation blocks converging survival signals by AKT and PIM kinases in lymphoma. J Exp Med. 2011;208:1799‐1807. 21859846 10.1084/jem.20110846 PMC3171093

[49] Beharry Z , Mahajan S , Zemskova M , et al. The Pim protein kinases regulate energy metabolism and cell growth. Proc Natl Acad Sci USA. 2011;108:528‐533. 21187426 10.1073/pnas.1013214108 PMC3021022

[50] Song JH , An N , Chatterjee S , et al. Deletion of Pim kinases elevates the cellular levels of reactive oxygen species and sensitizes to K‐Ras‐induced cell killing. Oncogene. 2015;34:3728‐3736. 25241892 10.1038/onc.2014.306 PMC4369476

[51] Diskin C , Palsson‐McDermott EM . Metabolic modulation in macrophage effector function. Front Immunol. 2018;9:270. 29520272 10.3389/fimmu.2018.00270 PMC5827535

