# Supplementary material for: Natural antiviral compound silvestrol modulates human monocyte‐derived macrophages and dendritic cells
Source: J Cell Mol Med. 2020 May 6;24(12):6988–99. doi: 10.1111/jcmm.15360 (PMC7267175; doi:10.1111/jcmm.15360)
Supplement: Supplementary file 1 — Fig S1 [file JCMM-24-6988-s001.pptx]

## Slide 1
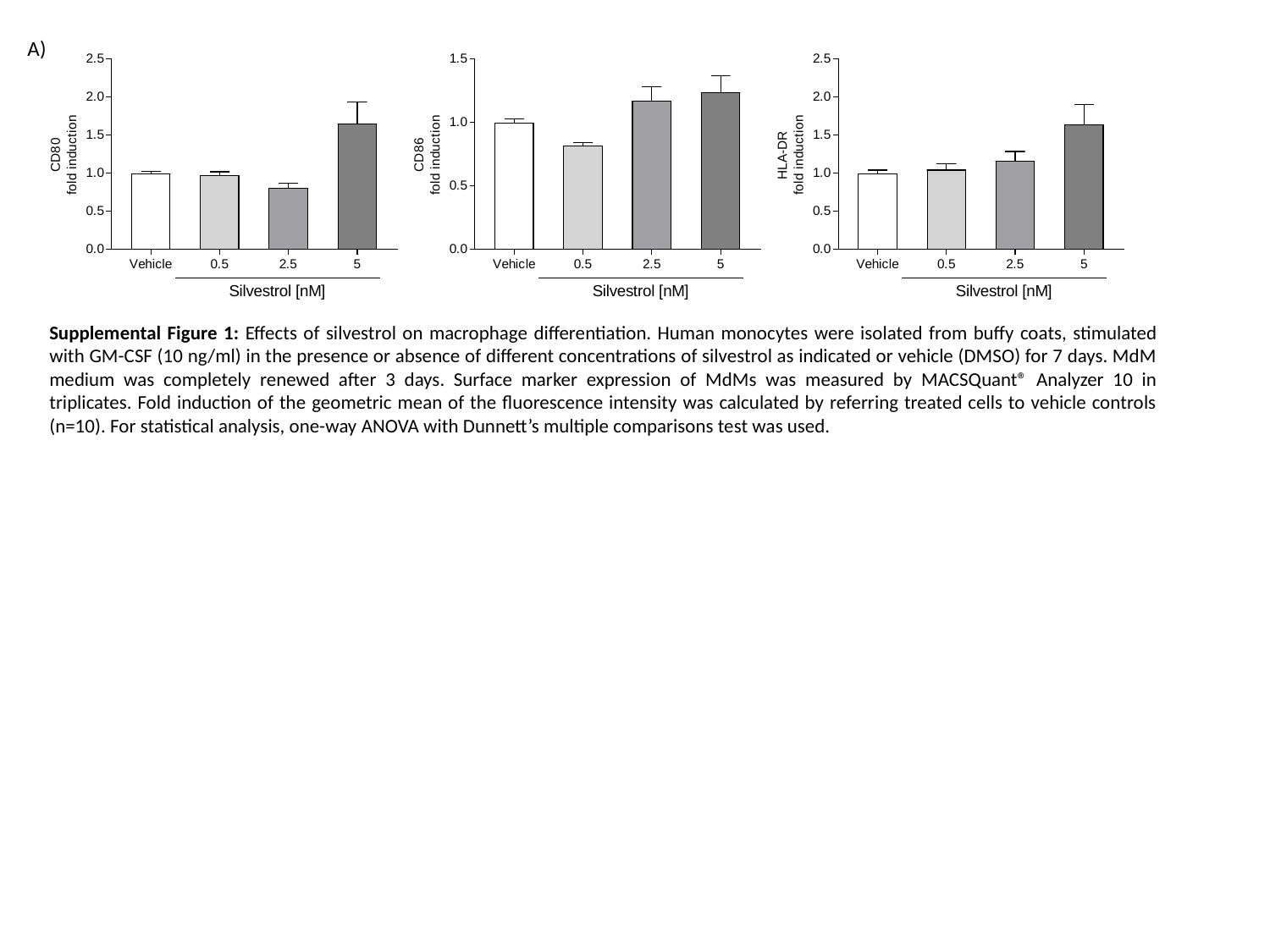

A)
Supplemental Figure 1: Effects of silvestrol on macrophage differentiation. Human monocytes were isolated from buffy coats, stimulated with GM-CSF (10 ng/ml) in the presence or absence of different concentrations of silvestrol as indicated or vehicle (DMSO) for 7 days. MdM medium was completely renewed after 3 days. Surface marker expression of MdMs was measured by MACSQuant® Analyzer 10 in triplicates. Fold induction of the geometric mean of the fluorescence intensity was calculated by referring treated cells to vehicle controls (n=10). For statistical analysis, one-way ANOVA with Dunnett’s multiple comparisons test was used.
