# Supplementary material for: Natural antiviral compound silvestrol modulates human monocyte‐derived macrophages and dendritic cells
Source: J Cell Mol Med. 2020 May 6;24(12):6988–99. doi: 10.1111/jcmm.15360 (PMC7267175; doi:10.1111/jcmm.15360)
Supplement: Supplementary file 2 — Fig S2 [file JCMM-24-6988-s002.pptx]

## Slide 1
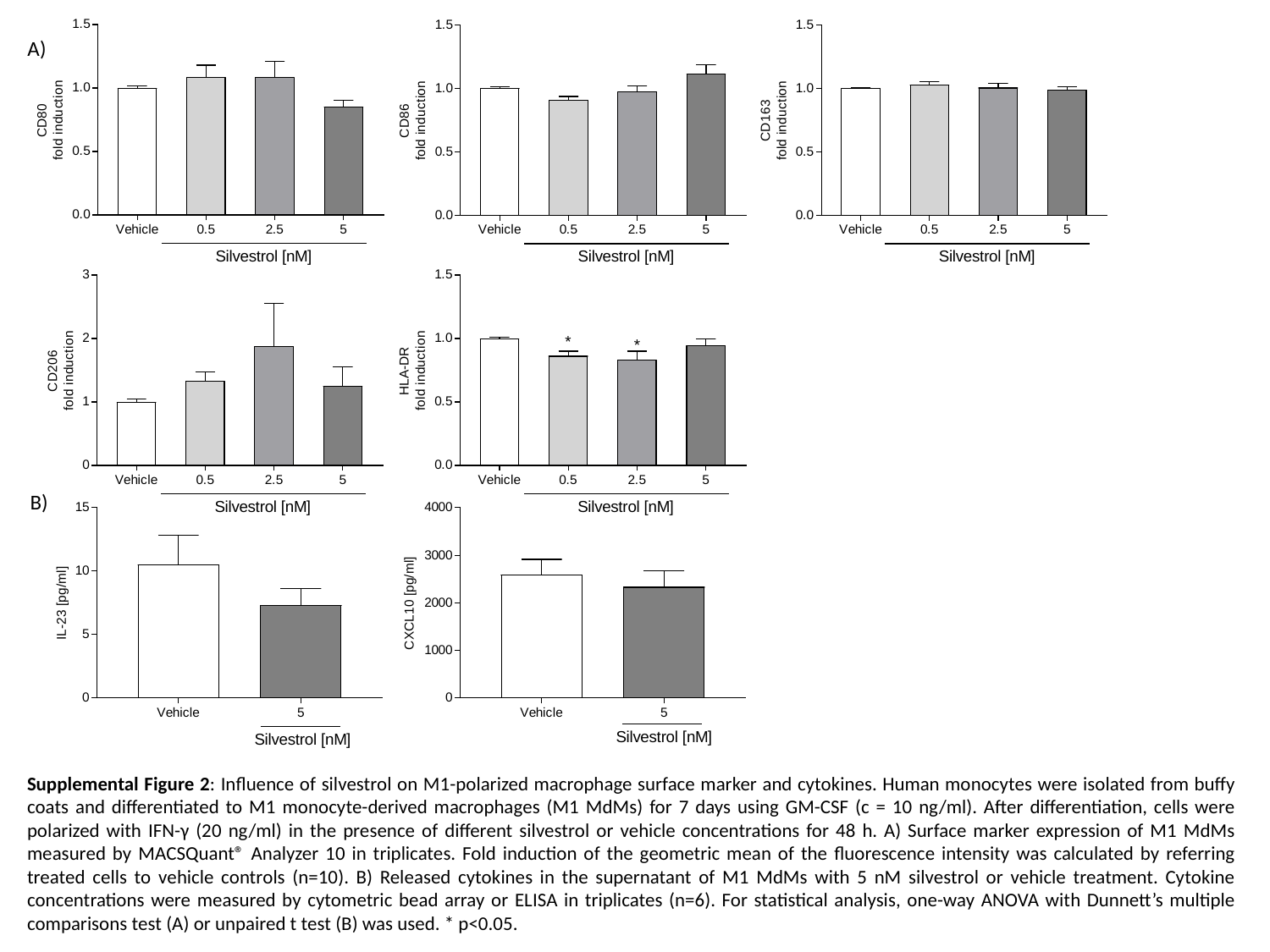

A)
B)
Supplemental Figure 2: Influence of silvestrol on M1-polarized macrophage surface marker and cytokines. Human monocytes were isolated from buffy coats and differentiated to M1 monocyte-derived macrophages (M1 MdMs) for 7 days using GM-CSF (c = 10 ng/ml). After differentiation, cells were polarized with IFN-γ (20 ng/ml) in the presence of different silvestrol or vehicle concentrations for 48 h. A) Surface marker expression of M1 MdMs measured by MACSQuant® Analyzer 10 in triplicates. Fold induction of the geometric mean of the fluorescence intensity was calculated by referring treated cells to vehicle controls (n=10). B) Released cytokines in the supernatant of M1 MdMs with 5 nM silvestrol or vehicle treatment. Cytokine concentrations were measured by cytometric bead array or ELISA in triplicates (n=6). For statistical analysis, one-way ANOVA with Dunnett’s multiple comparisons test (A) or unpaired t test (B) was used. * p<0.05.
