# Supplementary material for: Natural antiviral compound silvestrol modulates human monocyte‐derived macrophages and dendritic cells
Source: J Cell Mol Med. 2020 May 6;24(12):6988–99. doi: 10.1111/jcmm.15360 (PMC7267175; doi:10.1111/jcmm.15360)
Supplement: Supplementary file 3 — Fig S3 [file JCMM-24-6988-s003.pptx]

## Slide 1
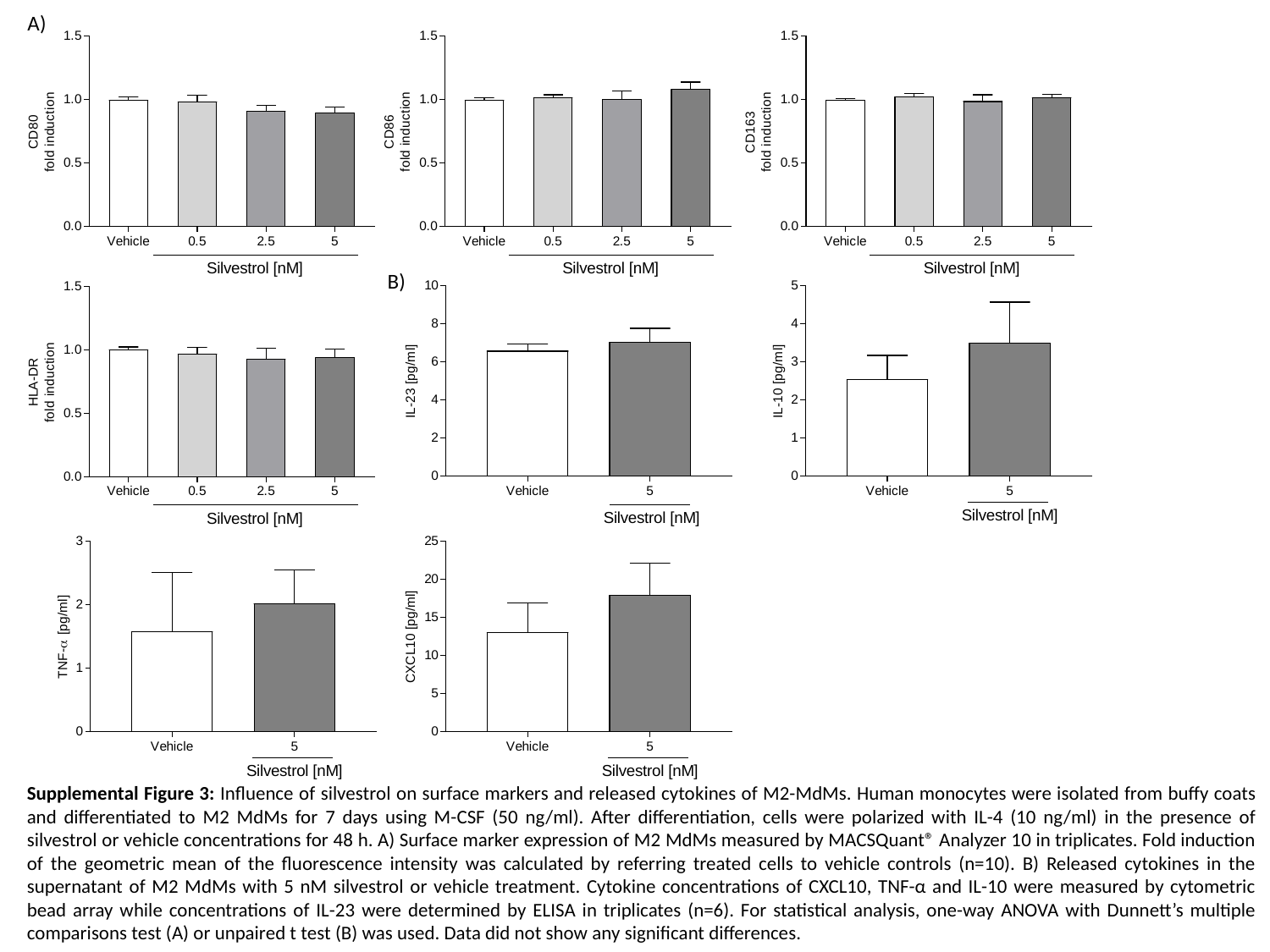

A)
B)
Supplemental Figure 3: Influence of silvestrol on surface markers and released cytokines of M2-MdMs. Human monocytes were isolated from buffy coats and differentiated to M2 MdMs for 7 days using M-CSF (50 ng/ml). After differentiation, cells were polarized with IL-4 (10 ng/ml) in the presence of silvestrol or vehicle concentrations for 48 h. A) Surface marker expression of M2 MdMs measured by MACSQuant® Analyzer 10 in triplicates. Fold induction of the geometric mean of the fluorescence intensity was calculated by referring treated cells to vehicle controls (n=10). B) Released cytokines in the supernatant of M2 MdMs with 5 nM silvestrol or vehicle treatment. Cytokine concentrations of CXCL10, TNF-α and IL-10 were measured by cytometric bead array while concentrations of IL-23 were determined by ELISA in triplicates (n=6). For statistical analysis, one-way ANOVA with Dunnett’s multiple comparisons test (A) or unpaired t test (B) was used. Data did not show any significant differences.
