# Supplementary material for: Natural antiviral compound silvestrol modulates human monocyte‐derived macrophages and dendritic cells
Source: J Cell Mol Med. 2020 May 6;24(12):6988–99. doi: 10.1111/jcmm.15360 (PMC7267175; doi:10.1111/jcmm.15360)
Supplement: Supplementary file 4 — Fig S4 [file JCMM-24-6988-s004.pptx]

## Slide 1
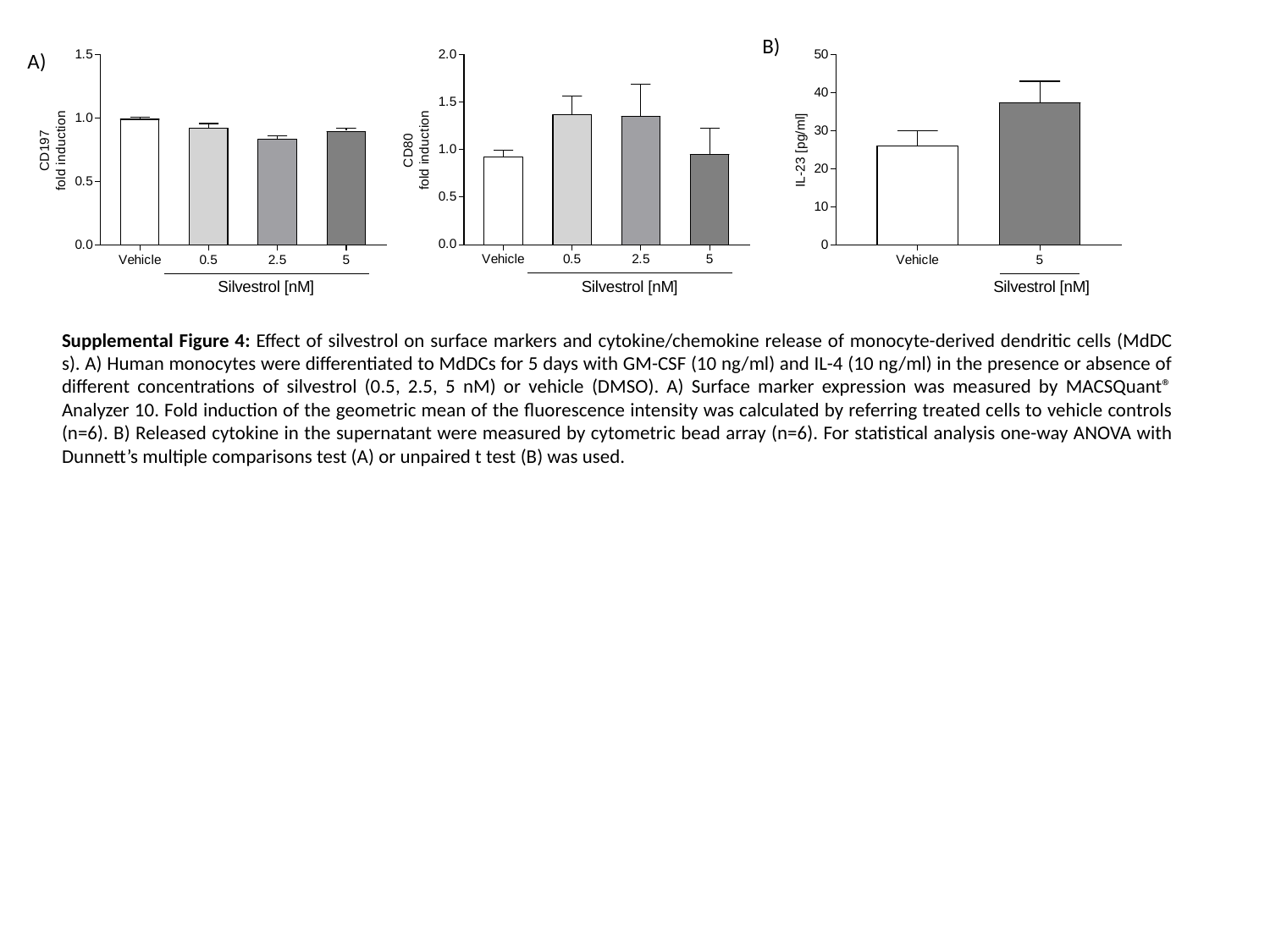

B)
A)
Supplemental Figure 4: Effect of silvestrol on surface markers and cytokine/chemokine release of monocyte-derived dendritic cells (MdDC s). A) Human monocytes were differentiated to MdDCs for 5 days with GM-CSF (10 ng/ml) and IL-4 (10 ng/ml) in the presence or absence of different concentrations of silvestrol (0.5, 2.5, 5 nM) or vehicle (DMSO). A) Surface marker expression was measured by MACSQuant® Analyzer 10. Fold induction of the geometric mean of the fluorescence intensity was calculated by referring treated cells to vehicle controls (n=6). B) Released cytokine in the supernatant were measured by cytometric bead array (n=6). For statistical analysis one-way ANOVA with Dunnett’s multiple comparisons test (A) or unpaired t test (B) was used.
