# Supplementary material for: Natural antiviral compound silvestrol modulates human monocyte‐derived macrophages and dendritic cells
Source: J Cell Mol Med. 2020 May 6;24(12):6988–99. doi: 10.1111/jcmm.15360 (PMC7267175; doi:10.1111/jcmm.15360)
Supplement: Supplementary file 5 — Fig S5 [file JCMM-24-6988-s005.pptx]

## Slide 1
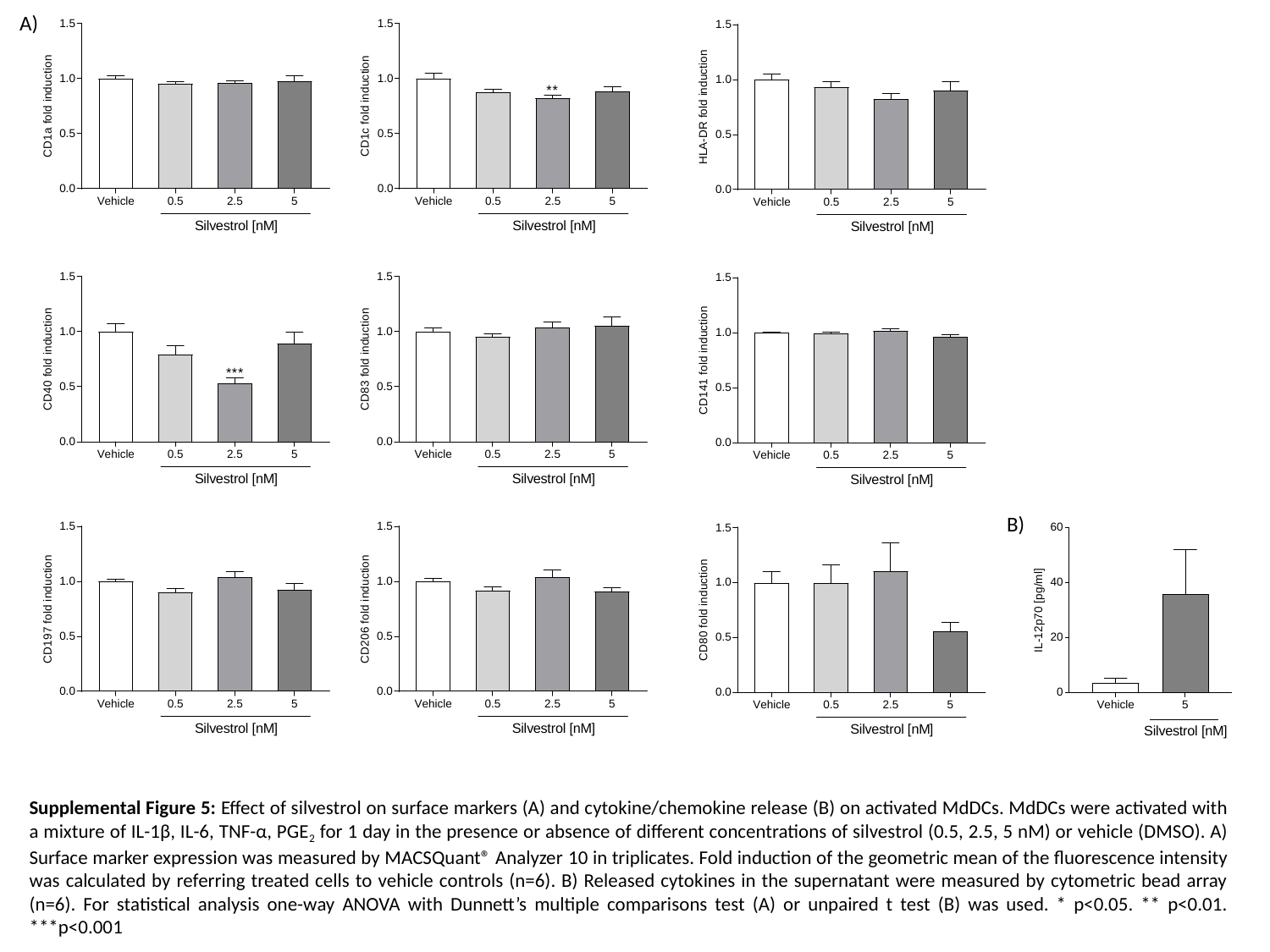

A)
B)
Supplemental Figure 5: Effect of silvestrol on surface markers (A) and cytokine/chemokine release (B) on activated MdDCs. MdDCs were activated with a mixture of IL-1β, IL-6, TNF-α, PGE2 for 1 day in the presence or absence of different concentrations of silvestrol (0.5, 2.5, 5 nM) or vehicle (DMSO). A) Surface marker expression was measured by MACSQuant® Analyzer 10 in triplicates. Fold induction of the geometric mean of the fluorescence intensity was calculated by referring treated cells to vehicle controls (n=6). B) Released cytokines in the supernatant were measured by cytometric bead array (n=6). For statistical analysis one-way ANOVA with Dunnett’s multiple comparisons test (A) or unpaired t test (B) was used. * p<0.05. ** p<0.01. ***p<0.001
